# Residual Adenoid Tissue After Conventional Adenoidectomy and the Role of Intraoperative Nasal Endoscopy: A Prospective Cohort Study

**DOI:** 10.3390/children12101393

**Published:** 2025-10-16

**Authors:** Goran Latif Omer, Aland Salih Abdullah, Sahand Soran Ali, Stefano Di Girolamo, Sveva Viola, Andrea Bravetti, Maria Grazia Maglie, Sara Maurantonio, Laura Borghesi, Othman Hussein Ahmed, Aso Khasraw Ahmed, Amanj Hamaamin Hamaamin, Hemn Hussein Othman, Giuseppe De Donato

**Affiliations:** 1 Department of Clinical Sciences, College of Medicine, University of Sulaimani, Sulaymaniyah 46001, Iraq; aland.05001758@univsul.edu.iq; 2 Department of Otorhinolaryngology, University of Rome Tor Vergata, 00133 Rome, Italy; stefano.di.girolamo@uniroma2.it (S.D.G.); svevaviola9@libero.it (S.V.); andrea-bravetti@libero.it (A.B.); mariagrazia.maglie@ptvonline.it (M.G.M.); sara.maurantonio@ptvonline.it (S.M.); laura.borghesi@ptvonline.it (L.B.); amanjhamaaminhamaamin@gmail.com (A.H.H.); gdedonato2@gmail.com (G.D.D.); 3 College of Pharmacy, American University of Iraq–Sulaimani, Sulaymaniyah 46001, Iraq; sahand.soran@auis.edu.krd; 4 Royal Hospital, Sulaymaniyah 46001, Iraq; oheartland@yahoo.com (O.H.A.); aso.k@yahoo.com (A.K.A.); hemnhussain88@gmail.com (H.H.O.)

**Keywords:** adenoidectomy, endoscopic surgery, pediatric sleep-disordered breathing, residual adenoid, microdebrider, revision surgery

## Abstract

**Highlights:**

**What are the main findings?**

- Residual adenoid tissue was detected in 61.8% of patients after conventional curettage adenoidectomy.
- Revision status and age ≥ 7.5 years were strong predictors of incomplete clearance.

**What is the implication of the main findings?**

- Intraoperative endoscopic assessment with completion resection significantly improves surgical outcomes.
- Primary endoscopic adenoidectomy is recommended for children ≥ 7.5 years and for revision cases.

**Abstract:**

**Background/Objectives**: Conventional curettage adenoidectomy is widely performed but may leave residual tissue in anatomically hidden nasopharyngeal areas. We evaluated the impact of age and revision status on residual adenoidal tissue after conventional adenoidectomy and assessed outcomes following endoscopic completion. **Methods**: A prospective cohort study included 178 patients undergoing conventional adenoidectomy followed by intraoperative nasal endoscopy. Residual tissue in the nasopharyngeal roof, Fossa of Rosenmüller, and around the Eustachian tube was resected using a microdebrider. Patients were categorized into four groups based on age (<9 or ≥9 years) and surgical history (primary vs. revision). Pediatric Sleep Questionnaire (PSQ) or STOP-BANG scores were collected pre- and postoperatively. Receiver operating characteristic (ROC) and logistic regression analyses were used to identify predictors of residual tissue. **Results**: Residual adenoid tissue was detected in 61.8% of patients after conventional adenoidectomy, highest among those ≥9 years undergoing revision (36.4%). Age ≥ 7.5 years and revision status predicted residual tissue (AUC = 0.71). Significant postoperative symptom improvement was observed (PSQ and STOP-BANG, *p* < 0.001). Complication rates were low (13.5%), with no recurrences reported. **Conclusions**: Conventional curettage often leaves residual adenoidal tissue in older and revision cases. Endoscopic completion improves outcomes. Primary endoscopic adenoidectomy is recommended for patients aged ≥7.5 years and those undergoing revision procedures.

## 1. Introduction

Adenoidectomy remains one of the most frequently performed surgical procedures in otolaryngology, primarily indicated for nasal obstruction, otitis media with effusion, recurrent otitis media, sleep-disordered breathing, eustachian tube dysfunction and chronic rhinosinusitis caused by adenoidal hypertrophy. Traditional curettage adenoidectomy, introduced over a century ago, is still widely practiced due to its simplicity, shorter operative time, and low cost [1,2,3,4]. However, the blind nature of this technique often results in incomplete removal of adenoidal tissue, particularly in the peritubal and superior nasopharyngeal recesses, leading to persistent symptoms and higher revision rates [2,5,6,7].

The advent of endoscopic techniques has significantly transformed adenoid surgery by allowing direct visualization of the nasopharynx, thereby improving the completeness and precision of tissue removal [5,7]. Microdebrider-assisted endoscopic adenoidectomy enables controlled tissue resection with minimal trauma to surrounding structures, reducing collateral injury and preserving Eustachian tube function [2,5,6]. Multiple comparative studies and randomized controlled trials have demonstrated that endoscopic approaches achieve superior clearance rates and fewer residual tissues compared to conventional methods, though they may be associated with slightly longer operative times and variable intraoperative blood loss [2,5,7,8,9].

Recent systematic reviews and meta-analyses have further supported the efficacy of endoscopic and powered techniques, showing significant reductions in residual adenoidal tissue and improved surgical accuracy, while highlighting the lack of a universally superior method due to variations in intraoperative bleeding, operative time, and postoperative complication [1]. Some studies have reported that powered techniques using microdebriders or coblation improve visualization and completeness of removal but may increase intraoperative blood loss compared to curettage [10,11]. Others found that endoscopic assistance can enhance outcomes even when using conventional curettes by minimizing the risk of retained tissue and Eustachian tube injury [5,9].

Given these evolving surgical modalities, there remains ongoing debate regarding the optimal approach for adenoidectomy. This study aims to evaluate the impact of patient age and history of prior adenoidectomy on clinical outcomes, while comparing conventional curettage and endoscopic microdebrider-assisted techniques. Importantly, the objective was not only to differentiate between these surgical approaches, but also to contribute to defining direct indications for endoscopic adenoidectomy. Since conventional adenoidectomy remains widely practiced worldwide due to its lower cost, this study seeks to provide evidence that may help guide surgeons in selecting cases where endoscopic control is particularly warranted.

## 2. Materials and Methods

### 2.1. Study Design and Setting

This study employed a prospective cohort design, in which 178 consecutive patients were enrolled between January and July 2025 at the first author’s clinic. All patients initially underwent conventional adenoidectomy. Subsequently, intraoperative nasal endoscopy was performed to assess the presence of residual adenoidal tissue. In cases where residual tissue was identified, endoscopic resection was carried out. Patient and surgical characteristics were subsequently analyzed in relation to both objective and subjective postoperative outcome measures.

### 2.2. Participants

Patients diagnosed with adenoid hypertrophy through anamnesis, physical examination, and nasal endoscopy were included, with or without a prior history of adenoidectomy. Exclusion criteria comprised patients with cleft palate, hematological disorders, age below 3 years, or general contraindications to surgery. For analytical purposes, patients were categorized into four groups based on age and surgical history:

- Group 1: Primary surgery in patients under 9 years of age (40 patients);
- Group 2: Secondary surgery in patients under 9 years of age (40 patients);
- Group 3: Primary surgery in patients aged 9 years or older (60 patients);
- Group 4: Secondary surgery in patients aged 9 years or older (38 patients).

Patients were divided into groups using 9 years of age as the cutoff, since this corresponded to the median age of the cohort and allowed balanced subgroup comparison. This threshold was therefore selected for pragmatic statistical grouping rather than as an established clinical benchmark.

### 2.3. Surgical Intervention

**Figure 1:**
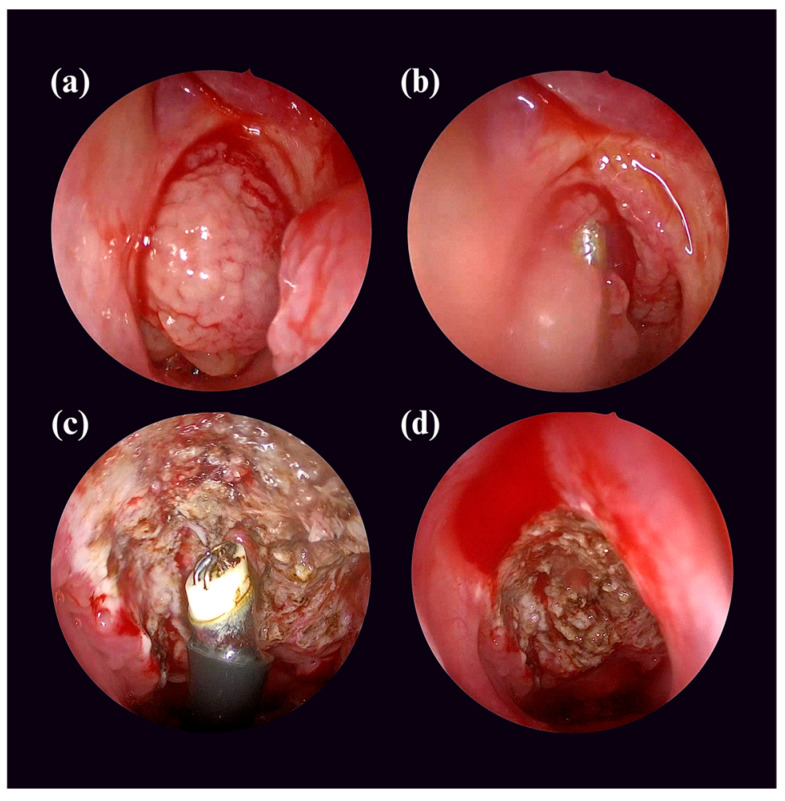
Intraoperative transnasal endoscopic images from a patient undergoing endoscopic clearance following conventional adenoidectomy to remove residual tissue. (**a**) Endoscopic view showing adenoid tissue extending into and obstructing the right choana before conventional adenoidectomy. (**b**) Use of a microdebrider for endoscopic removal of the remaining adenoid tissue. (**c**) Application of a coblation device to achieve hemostasis after resection of the residual tissue. (**d**) Endoscopic view of the nasopharynx at the conclusion of the procedure, showing a clear surgical field.

All procedures were performed under general anesthesia with orotracheal intubation and the patient positioned supine with the neck gently extended. Before adenoidectomy, the adenoid tissue was examined transorally and endoscopically through the nose (Figure 1a). Then, conventional adenoidectomy was carried out using a Thompson adenoid curette introduced transorally. Digital palpation was performed prior to curettage to assess the size and extent of adenoidal tissue and to help direct the instrument toward the nasopharyngeal roof and posterior choana. Hemostasis was achieved using standard packing. Electrocautery was not used in these cases, as it is typically applied only on the tonsillar bed after tonsillectomy and cannot reach the adenoids; instead, hemostasis was supported by coblation when required.

Following conventional curettage, every patient underwent intraoperative endoscopic evaluation of the nasopharynx using a 0° rigid nasal endoscope introduced transnasally. The inspection focused on areas commonly associated with incomplete clearance, including the peritubal recess near the Eustachian tube orifice, the nasopharyngeal roof, and the Fossa of Rosenmüller. This protocol was identical for both primary and revision cases, as all patients underwent curettage first, followed by endoscopic assessment. In cases where residual adenoidal tissue was identified, completion adenoidectomy was performed under direct endoscopic visualization. This was achieved transorally using a microdebrider to remove the remnant tissue (Figure 1b), while a coblation probe was applied selectively to secure hemostasis when necessary (Figure 1c). Both instruments were not used concurrently for tissue removal, but rather in complementary roles: the microdebrider for resection and coblation exclusively for hemostasis. A final endoscopic check was performed to confirm complete clearance (Figure 1d) and ensure that the Eustachian tube openings and nasopharyngeal mucosa were intact.

### 2.4. Variables

1 Anamnesis data, including symptoms and history of previous adenoidectomy.
2 Preoperative clinical findings, including nasal endoscopy and general examination results.
3 Subjective measures of symptom severity, assessed both preoperatively and postoperatively:
  - Pediatric Sleep Questionnaire (PSQ) for the evaluation of obstructive sleep apnea (OSA) in patients under 18 years of age [12].
  - STOP-BANG score for OSA screening in patients aged 18 years and older [13].
4 Operative details, including the type of surgical procedure performed, whether concomitant tonsillectomy was conducted, Parikh et al.’s adenoid hypertrophy grading [14], and other relevant intraoperative observations.
5 All patients were followed postoperatively at 1 week, 1 month, and 2 months. Each visit included clinical examination, evaluation of symptom resolution, and documentation of complications such as bleeding, infection, or otalgia. Parents or patients were questioned regarding persistence or recurrence of nasal obstruction and otologic symptoms. Symptom severity was assessed at each visit using the Pediatric Sleep Questionnaire (PSQ) for patients younger than 18 years and the STOP-Bang questionnaire for patients aged 18 years or older. Both instruments were re-administered at the final 2-month follow-up to evaluate postoperative improvement in sleep-disordered breathing. Nasal endoscopy was not performed routinely, but was selectively conducted in patients with persistent symptoms to rule out residual or recurrent adenoid tissue.

### 2.5. Ethical Approval and Participation Consent

This study was approved by the Ethics Committee of the University of Sulaimani, College of Medicine. Approval Number 165, in their 15th meeting on 17 August 2025. Written informed consent was obtained from all participants (or guardians).

### 2.6. Statistical Analysis

All statistical analyses were performed using JASP (version 0.19.3) and R (version 4.5.0). Continuous variables were assessed for normality using the Shapiro–Wilk test and were found to be non-normally distributed. These variables were summarized as medians with interquartile ranges (IQRs), while categorical variables were presented as frequencies and percentages. Preoperative and postoperative Pediatric Sleep Questionnaire (PSQ) scores in children and STOP-BANG scores in adults were compared using the Wilcoxon signed-rank test for paired, non-parametric data. Associations between categorical variables (e.g., patient groups, adenoid grading, age group, tissue surface characteristics) and postoperative complications or clearance rates were examined using the Chi-square test of independence or Fisher’s exact test when expected cell counts were <5. Effect sizes were reported as Cramér’s V or φ for categorical associations, relative risks (RR) for binary outcomes, and median differences for nonparametric comparisons, each with 95% confidence intervals. A *p*-value < 0.05 was considered statistically significant for all analyses.

## 3. Results

A total of 178 patients underwent conventional adenoidectomy followed by intraoperative nasal endoscopy to assess the need for additional endoscopic resection. Patients were categorized into 4 groups: primary surgery under 9 years (Group 1, n = 40), secondary surgery under 9 years (Group 2, n = 40), primary surgery 9 years or older (Group 3, n = 60), and secondary surgery 9 years or older (Group 4, n = 38). The median age of the cohort was 9 years (interquartile range [IQR], 6–12), and 48.3% were female. Secretory otitis media was the most common comorbidity (16.9%), while 81.5% of patients had no comorbid conditions (Table 1).

**Figure 2:**
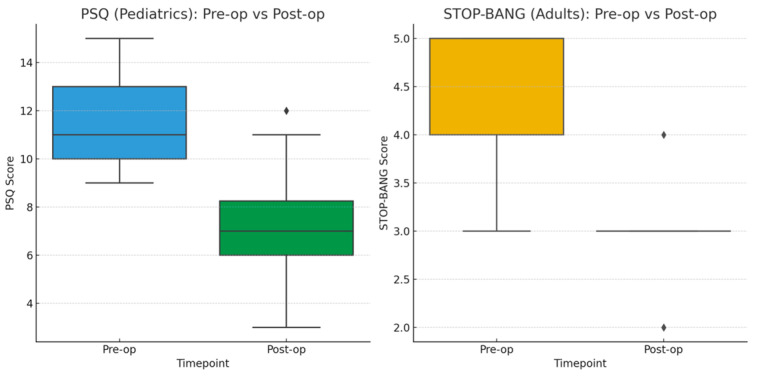
Paired boxplots comparing preoperative and postoperative scores for (**Left Panel**) Pediatric Sleep Questionnaire (PSQ) in children and (**Right Panel**) STOP-BANG scores in adults following adenoidectomy. For pediatric patients (**Left Panel**), median PSQ scores significantly decreased from 11 (IQR 10–13) preoperatively to 7 (IQR 6–8) postoperatively (Wilcoxon signed-rank test, W = 12,880, z = 10.97, *p* < 0.001). For adult patients (**Right Panel**), STOP-BANG scores improved from a median of 5 (IQR 4–5) to 3 (IQR 3–3) postoperatively (Wilcoxon signed-rank test, W = 171, z = 3.72, *p* < 0.001). Both analyses demonstrate significant postoperative improvement in sleep-disordered breathing symptoms.

Symptom scores improved significantly after surgery. Among pediatric patients, Pediatric Sleep Questionnaire (PSQ) scores decreased from a median of 11 (IQR, 10–13) preoperatively to 7 (IQR, 6–8) postoperatively (Wilcoxon signed-rank test: W = 12,880, z = 10.97, *p* < 0.001). In adults, STOP-BANG (Snoring, Tiredness, Observed Apnea, Pressure, Body Mass Index, Age, Neck circumference, Gender) scores improved from a median of 5 (IQR, 4–5) to 3 (IQR, 3–3) (W = 171, z = 3.72, *p* < 0.001) (Figure 2). Nasal obstruction was present in all patients preoperatively and resolved completely after the procedure. Intraoperative and postoperative variables are summarized in Table 2.

Intraoperative endoscopic assessment frequently identified residual adenoid tissue in the hidden areas (nasopharyngeal roof, eustachian tube region, and Fossa of Rosenmüller) following conventional adenoidectomy (61.8%). Clearance rates in these regions differed significantly between groups (χ^2^[3, N = 178] = 36.05, *p* < 0.001, Cramér’s V = 0.45, 95% CI, 0.01–0.65). The need for additional endoscopic resection was most pronounced in Group 4, where 36.4% required clearance of residual tissue. In contrast, Group 1 had the lowest rate, with only 9.1% requiring additional resection. Adenoid surface characteristics were also associated with residual tissue; uneven surfaces were significantly more common in secondary surgeries (χ^2^[1, N = 178] = 23.83, *p* < 0.001, φ = 0.38, 95% CI, 0.25–0.51) (Table 3).

Postoperative complications occurred in 13.5% of patients, with bleeding reported in 3.4% and otalgia in 10.1%. The highest complication rate was observed in Group 1, where 40% experienced at least 1 complication compared with ≤10% in other groups (χ^2^[3, N = 178] = 34.16, *p* < 0.001, risk difference = 0.21, 95% CI, 0.11–0.31). Adenoid grade was also associated with complications, with Grade 4 patients having higher rates of otalgia than Grade 3 (χ^2^[2, N = 178] = 6.95, *p* = 0.031, Cramér’s V = 0.20, 95% CI, 0.08–0.33). Younger patients (<9 years) had more complications than older patients (χ^2^[1, N = 178] = 14.78, *p* < 0.001, relative risk = 6.13, 95% CI, 2.18–17.19) (Table 4). No recurrence was observed.

**Figure 3:**
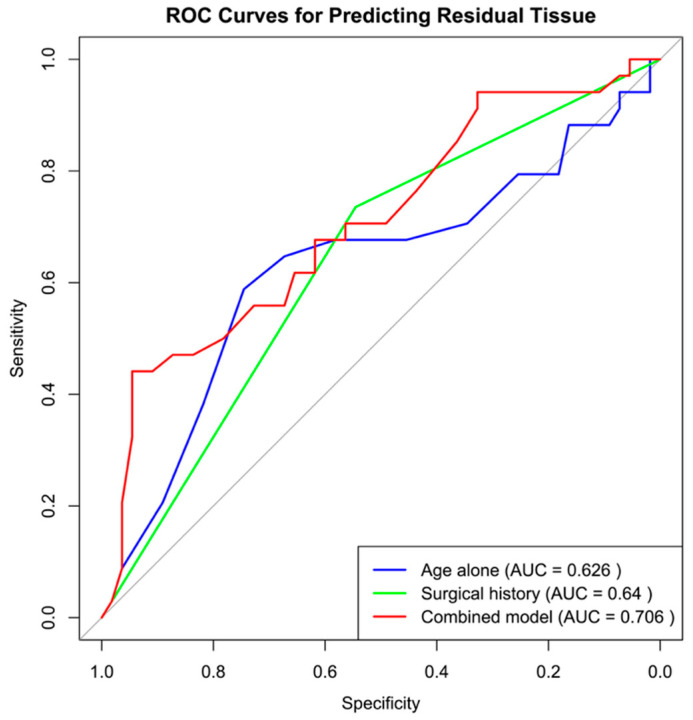
Receiver operating characteristic (ROC) curves showing the predictive value of age alone (blue), surgical history (green), and the combined logistic regression model (red) for detecting residual adenoid tissue in the Fossa of Rosenmüller, area around the Eustachian tube and nasopharyngeal roof. The area under the curve (AUC) was 0.626 for age alone, 0.64 for surgical history, and 0.706 for the combined model. Optimal cutoff values based on the Youden Index were 7.5 years for age, 1.5 for surgical history (primary vs. revision), and 0.55 for the combined model.

Receiver operating characteristic (ROC) analysis was performed to evaluate whether age predicted residual tissue in hidden areas. Age alone yielded an area under the curve (AUC) of 0.63 (*p* = 0.02, 95% CI, 0.53–0.72), with an optimal cutoff at 7.5 years. A logistic regression model including both age and revision status showed that revision status was a strong predictor (OR, 3.82; 95% CI, 1.94–7.50; *p* < 0.001), while age approached significance (OR per year, 1.04; 95% CI, 1.00–1.08; *p* = 0.073). The combined model achieved an AUC of 0.71 (95% CI, 0.61–0.80), higher than age alone, although DeLong’s test indicated this improvement was not statistically significant (ΔAUC, 0.08; 95% CI, –0.03 to 0.19; *p* = 0.16) (Figure 3). Additional analyses were performed to test whether the effect of age differed by sex and whether non-linear effects of age could be identified. The age-by-sex interaction was not significant, indicating that the influence of age on outcomes was similar in boys and girls. Modeling age as a continuous predictor using restricted cubic splines did not significantly improve model fit, suggesting that the relationship between age and complication risk was adequately captured by a linear trend. Among patients requiring endoscopic clearance, 69% (76 of 110) had adenoid tissue obstructing the choana when examined transnasally with endoscopy.

## 4. Discussion

Adenoidectomy is among the most commonly performed pediatric otolaryngology procedures worldwide and is traditionally carried out using blind curettage [15,16]. While this method remains popular because of its simplicity and accessibility, several studies have shown that it often fails to achieve complete removal of adenoid tissue. Residual tissue is frequently left in challenging anatomical regions such as the nasopharyngeal roof and the Fossa of Rosenmüller, areas that are difficult to visualize and reach with conventional curettage [17,18]. This incomplete clearance is clinically important because it can result in persistent nasal obstruction, ongoing sleep-disordered breathing, and, in some cases, the need for revision surgery [19,20]. The findings of this prospective cohort study reinforce these known limitations. Intraoperative endoscopic assessment revealed that residual tissue was most often located in the nasopharyngeal roof and Fossa of Rosenmüller, confirming that blind curettage provides limited access to these areas. Similar findings have been reported in previous research, where revision rates varied by surgical technique but incomplete clearance was consistently more common with traditional curettage [15]. A systematic analysis of more than 1000 patients concluded that residual adenoid tissue often persists in hidden recesses inaccessible to blind instruments [17]. Microdebrider-assisted adenoidectomy has been shown to leave significantly fewer remnants compared with conventional approaches, and other studies have demonstrated that endoscopic assistance enables near-complete removal, whereas conventional techniques left residual tissue in up to 38% of cases [16,18,19,20]. The rate of incomplete clearance observed in the present study aligns closely with these findings, further supporting the role of visualization in achieving comprehensive adenoid removal. Another important consideration is that revision adenoidectomy may also be indicated in patients with persistent otitis media with effusion, reflecting the close association between adenoid size and middle ear ventilation [4].

Postoperative symptom improvement in this cohort, including marked reductions in Pediatric Sleep Questionnaire and STOP-BANG scores, is consistent with reports that endoscopic techniques lead to superior resolution of nasal obstruction and sleep-disordered breathing. Enhanced visualization during surgery has also been shown to improve Eustachian tube function and reduce persistent symptoms [21,22,23]. In the present study, complete symptom resolution was achieved, but frequently only after endoscopic completion of tissue removal, underscoring the importance of thorough clearance.

In this cohort, the most frequent postoperative complications were bleeding and otalgia, both of which were mild and self-limiting. Other potential complications of adenoidectomy, including velopharyngeal insufficiency, nasopharyngitis, hemotympanum, torticollis, and Grisel syndrome, were specifically assessed during follow-up, but none of the patients in this series developed these events. Postoperative bleeding occurred in 3.4% of patients and otalgia in 10.1%. All bleeding events were mild, occurred in the early postoperative period, and were managed conservatively without the need for surgical re-exploration. This bleeding rate is higher than the <1% typically reported in systematic reviews of endoscopic adenoidectomy [24]. The difference may be attributable to the relatively high proportion of revision cases and younger patients included in this cohort, as well as the absence of electrocautery for hemostasis. Otalgia, observed in 10.1% of patients, was a mild and self-limiting symptom consistent with postoperative discomfort described in other adenoidectomy series [15]. Importantly, no recurrences were observed during follow-up, likely because endoscopic verification was routinely used to ensure total resection. Earlier studies have suggested that intraoperative visualization significantly reduces the risk of adenoid regrowth [17,19].

Although earlier studies have compared conventional and endoscopic adenoidectomy techniques, none have proposed a clear age threshold to guide surgical approach. Some research has noted differences in outcomes between younger and older children [14,25,26,27], reporting that as children grow, adenoid tissue becomes more fibrotic and extends further laterally, making complete removal more challenging. In this study, nine years of age was initially selected as a practical cutoff to define older children, as this corresponded to the median age of the current cohort. However, statistical analysis showed that an age threshold of 7.5 years offered better predictive accuracy for identifying patients at higher risk of residual tissue. Logistic regression further demonstrated that revision status was an independent and strong predictor, and when combined with age, the model achieved improved discriminative performance (AUC 0.71). Based on these findings, it is recommended that children aged 7.5 years or older, as well as those undergoing revision adenoidectomy, undergo endoscopic adenoidectomy rather than relying solely on blind curettage. Finally, the authors have also seen that most patients who have adenoid tissue obstructing the choana when examined endoscopically through the nose, require endoscopic resection to make sure there is no remnants left and the tissue is removed in its entirety. These recommendations should be validated by larger, multicenter studies to establish a definitive age-based guideline. The limitations of this study included follow-up at 1 week, 1 month, and 2 months, which allowed for reliable assessment of early symptom resolution and complications. However, longer follow-up could provide additional insights into long-term recurrence and late complications. Another limitation lies in the monitored parameters, as the study did not incorporate a comparison of adenoid size in relation to the choanae or the tubal tori, which may influence both symptoms and surgical outcomes. Additionally, outcomes were not stratified by obstruction grade or surgical indication (nasal obstruction versus otitis media with effusion), which may have masked clinically relevant differences. Furthermore, polysomnography was not performed, instead, sleep-disordered breathing was evaluated using the Pediatric Sleep Questionnaire in children and STOP-Bang in adults, where its role was limited to symptom severity in secondary analyses. The absence of objective sleep studies reflects the limited availability and high cost of polysomnography in the region, and this should be considered when interpreting the sleep-related findings.

## 5. Conclusions

This prospective cohort study demonstrates that conventional curettage adenoidectomy frequently leaves residual tissue, particularly in older children and those undergoing revision surgery. Intraoperative nasal endoscopy identified hidden remnants in more than 60% of cases, most commonly in the nasopharyngeal roof and peritubal recesses. Importantly, our analysis established that both revision status and age ≥ 7.5 years are independent predictors of incomplete clearance, thereby providing quantitative thresholds that can guide surgical decision-making. While residual tissue was more common in larger adenoids, incomplete clearance was also observed in smaller ones, underscoring that all adenoidectomies may merit endoscopic control to ensure completeness and safety. Instead, they underscore the clinical value of tailoring the surgical approach according to age and revision history. Based on these results, we recommend primary endoscopic adenoidectomy for patients aged 7.5 years or older and for those requiring revision procedures. Larger, multicenter studies with long-term follow-up are warranted to validate these recommendations and further refine evidence-based guidelines for adenoidectomy.

## Figures and Tables

**Table 1:** Baseline Demographics and Preoperative Clinical Variables.

Variables	N (%)
Patient History	
Nasal Obstruction	178 (100%)
Otalgia	28 (15.7%)
Rhinorrhea	86 (48.3%)
Nasal Tone	122 (68.5%)
Postnasal Discharge	98 (55.1%)
History of Adenotonsillectomy	84 (47.2%)
Nasal Obstruction	178 (100%)
Otalgia	28 (15.7%)
Rhinorrhea	86 (48.3%)
Nasal Tone	122 (68.5%)
Comorbidities	
Secretory Otitis Media	34 (19.1%)
Chronic Suppurative Otitis Media	4 (2.2%)
Total Patients	178 (100%)

**Table 2:** Intraoperative and Postoperative Variables.

Variables	N (%)
Adenoid Tissue Surface	
Smooth	74 (41.6%)
Uneven	104 (58.4%)
Parikh Grading Scale	
Grade 3	54 (30.3%)
Grade 4	124 (69.7%)
Intraoperative Detail	
Endoscopic Clearance from Hidden Areas ^1^ After Conventional Adenoidectomy	110 (61.8%)
Soft Palate Elevation	178 (100%)
Postoperative Complications	
Bleeding	6 (3.4%)
Otalgia	18 (10.1%)
Postoperative Nasal Obstruction	0 (0%)
Recurrence of Adenoid Tissue	0 (0%)
Total Patients	178 (100%)

^1^ Hidden Areas: Nasopharyngeal roof, area around the Eustachian tube, and/or the Fossa of Rosenmuller.

**Table 3:** The relationship between the patient groups with certain intra- and postoperative variables.

		Group 1	Group 2	Group 3	Group 4	*p*-Value
Endoscopic Clearance from Hidden Areas ^1^ After Conventional Adenoidectomy	Yes	10 (9.1%)	26 (23.6%)	34 (30.9%)	40 (36.4%)	<0.001
	No	30 (44.1%)	14 (20.6%)	4 (5.9%)	20 (29.4%)	
Postoperative Complications	None	24 (15.6%)	36 (23.4%)	60 (38.9%)	34 (22.1%)	<0.001
	Bleeding	6 (100%)	0 (0%)	0 (0%)	0 (0%)	
	Otalgia	10 (55.6%)	4 (22.2%)	0 (0%)	4 (22.2%)	
Adenoid Tissue Surface	Smooth	26 (35.1%)	16 (21.6%)	32 (21.6%)	0 (43.3%)	<0.001
	Uneven	14 (13.5%)	24 (23.1%)	28 (26.9%)	38 (36.5%)	

^1^ Hidden Areas: Nasopharyngeal roof, area around the Eustachian tube, and/or the Fossa of Rosenmuller.

**Table 4:** Association Between Adenoid Grading Scale and Postoperative Complications.

		Postoperative Complication			*p*-Value
		No Complications	Bleeding	Otalgia	
Parikh Grading Scale	Grade 3	48 (31.2%)	4 (66.7%)	2 (11.1%)	0.031
	Grade 4	106 (68.8%)	2 (33.3%)	16 (88.9%)	

## Data Availability

The datasets generated and analyzed during the current study are available from the corresponding author on reasonable request.

## Abbreviations

The following abbreviations are used in this manuscript:

PSQ	Pediatric Sleep Questionnaire
STOP-BANG	Snoring, Tiredness, Observed apnea, Pressure, Body-mass index, Age, Neck circumference, Gender (OSA screening tool)
OSA	Obstructive Sleep Apnea
IQR	Interquartile Range
ROC	Receiver Operating Characteristic
AUC	Area Under the Curve
CI	Confidence Interval (95% CI)
RR	Relative Risk
OR	Odds Ratio
χ^2^	Chi-square statistic
W	Wilcoxon signed-rank test statistic
z	Standard normal z-score
ΔAUC	Difference in area under the curve
